# Clopidogrel and Aspirin versus Aspirin Alone for Stroke Prevention: A Meta-Analysis

**DOI:** 10.1371/journal.pone.0135372

**Published:** 2015-08-13

**Authors:** Shuai Tan, Xiaojuan Xiao, Hanyu Ma, Zhaohui Zhang, Jiangbo Chen, Lei Ding, Shenping Yu, Rulin Xu, Shiliang Yang, Xinyi Huang, Hua Hong

**Affiliations:** 1 Department of Neurology, The First Affiliated Hospital, Sun Yat-sen University, Guangzhou 510080, People's Republic of China; 2 Department of Radiology, The First Affiliated Hospital, Sun Yat-sen University, Guangzhou 510080, People's Republic of China; 3 Department of Pathology, The First Affiliated Hospital, Sun Yat-sen University, Guangzhou 510080, People's Republic of China; 4 Department of Pancreato-Biliary Surgery, The First Affiliated Hospital, Sun Yat-sen University, Guangzhou 510080, People's Republic of China; 5 Department of Interventional Radiology, The First Affiliated Hospital, Sun Yat-sen University, Guangzhou 510080, People's Republic of China; 6 Department of Thoracic Surgery, The First Affiliated Hospital, Sun Yat-sen University, Guangzhou 510080, People's Republic of China; 7 Department of Cardiology, The First Affiliated Hospital, Sun Yat-sen University, Guangzhou 510080, People's Republic of China; Royal College of Surgeons, IRELAND

## Abstract

**Background and Purpose:**

Antiplatelet therapy is widely used for the primary or secondary prevention of stroke. Drugs like clopidogrel have emerged as alternatives for traditional antiplatelet therapy, and dual therapy with clopidogrel and aspirin is of particular interest. We conducted this meta-analysis to systematically review studies about dual therapy comparing monotherapy with aspirin alone.

**Methods:**

Randomized controlled trials were searched in PubMed (1966-May, 2015), EMBASE (1947-May, 2015), the Cochrane Central Register of Controlled Trials (CENTRAL) (1948-May, 2015), WHO International Clinical Trial (ICTRP) (2004-May, 2015), China Biology Medicine disc (CBM disc) (1978-May, 2015) and were included into the final analysis according to the definite inclusion criteria mentioned in the study selection section. Risk ratio (RR) was pooled with 95% confidence interval (CI) for dichotomous data. The heterogeneity was considered significant if the χ^2^ test was significant (P value < 0.10) or the I^2^ > 50.00%. Subgroup analyses were carried out on the long and short time periods, the race and region.

**Results:**

We included 5 studies involving 24,084 patients. A pooled analysis showed that dual therapy with clopidogrel and aspirin had a lower stroke incidence than monotherapy in both the short term and long term (RR = 0.69, 95% CI: 0.59–0.82, P <0.05; RR = 0.84, 95% CI: 0.72–0.98, P = 0.03, respectively). With regard to safety, dual therapy had a higher risk of bleeding than monotherapy for both periods (RR = 1.51, 95% CI: 1.03–2.23, P = 0.04; RR = 1.54, 95% CI: 1.32–1.79, P<0.05, respectively).

**Conclusions:**

Dual therapy with clopidogrel and aspirin could be a preferable choice to prevent stroke in patients who have had a previous stroke or transient ischemic attack, as well as those who are at high risk for stroke. And the effect of dual therapy seems to be more obvious for short-term. However, it is associated with a higher risk of bleeding.

## Introduction

Stroke has high mortality, disability, and recurrence rates and is one of the leading causes of death worldwide [1,2,3]. Subjects who experience a transient ischemic attack (TIA) have 5% and 10% incidences of having a stroke within 48 hours and 3 months, respectively [4,5]. A specific type is the minor stroke, which has been focused on after being first reported by Epstein and Boas in 1955 [6,7]. It is defined by: (1) a score of 0–1 on the National Institutes of Health Stroke Scale (NIHSS) plus a score of 0 in consciousness items or (2) an NIHSS score <3 [8].

Aspirin is now the first-line antiplatelet drug, and its effectiveness in reducing stroke recurrence has been demonstrated in a large number of clinical trials [9]. As an alternative, clopidogrel can reach its peak antiplatelet effect within 3 hours after the administration of a first dosage of 300 mg (or 7 days to reach peak with 75 mg/d). Studies evaluating this agent often ask the patients to take it for 28 days, although this has been associated with adverse effects such as mucocutaneous hemorrhage [10,11,12,13,14].

Several studies have been published describing the combination of clopidogrel and aspirin versus aspirin alone for ischemic stroke prevention, but the results were variable. Here, we analyzed eligible randomized studies comparing the effects and safety of clopidogrel and aspirin versus aspirin alone to clarify the effects and possibly guide clinical use.

## Materials and Methods

### Search Strategy

We identified eligible trials by performing electronic searches of PubMed (1966 to May, 2015), EMBASE(1947 to May, 2015), the Cochrane Central Register of Controlled Trials (CENTRAL) (1948 to May, 2015), WHO International Clinical Trial (ICTRP) (2004 to May, 2015) and China Biology Medicine disc (CBMdisc) (1978 to May, 2015) using the search items related to aspirin, clopidogrel and stroke (i.e., “Aspirin” or “Acetylsalicylic Acid” or “Acid, Acetylsalicylic” or “2-(Acetyloxy)benzoic Acid” or “Acylpyrin” or “Colfarit” or “Ecotrin” or “Endosprin” or “Magnecyl” or “Micristin” or “Polopirin” or “Polopiryna” or “Solupsan” or “Zorprin” or “Acetysal” or “clopidogrel” or “clopidogrel napadisilate” or “Iscover” or “clopidogrel hydrochloride” or “clopidogrel-Mepha” or “Plavix” or “SR 25989” or “clopidogrel besylate” or “clopidogrel Sandoz” or “clopidogrel bisulfate” or “PCR 4099” or “PCR-4099” or “clopidogrel hydrogensulfate” or “Plavix” AND "Stroke" or "Hypoxia-Ischemia, Brain" or "Intracranial Arterial Diseases" or "Cerebral Arterial Diseases" or "Intracranial Embolism and Thrombosis" or "Carotid Artery Diseases" or "Carotid Artery Thrombosis" or "Ischemic Attack, Transient" or “stroke*” or “apoplexy*” or “cerebral vasc*” or “cerebrovasc*” or “CVA” or “(transient ischemic attack*)” or “(transient ischaemic attack)” or “TIA*”). We limited the languages to English and Chinese and got ready to retrieve potentially relevant studies from the references lists to identify studies that may have missed. And no more studies met our standards finally. The time period included was from the establishment of the databases to May, 2015.

### Study Selection

Studies were included if (1) patients were older than 18 years old and had either had (a) a noncardiogenic stroke within 3 days, (b) TIA, (c) minor stroke; or (d) a high risk of stroke due to atherosclerosis or hyperlipidemia. (2) Only randomized controlled trials (RCTs) were considered. (3) The included studies primarily compared a combination of clopidogrel and aspirin versus aspirin alone, but we also included studies that compared other drugs but also had groups using clopidogrel and aspirin or aspirin alone.

The participant exclusion criteria were mainly based on the included studies, but we also excluded studies that (1) focused on strokes caused by cardiogenic thrombus, (2) did not assess any of the outcomes, and (3) lacked primary data.

### Data Extraction and Quality Assessment

All data from eligible studies were independently abstracted by two investigators (Hanyu Ma and Zhaohui Zhang) according to the aforementioned inclusion criteria. Discrepancies were resolved by discussion with a third investigator (Jiangbo Chen) and by referencing the original report. Recorded data variables were the author, publication year, sample size, baseline participant characteristics, treatment dosage, and treatment duration. Then we (Shuai Tan, Xiaojuan Xiao, Lei Ding) extracted the data assessed the quality of included studies according to the Cochrane risk of bias tools (www.cochrane.org/training/cochrane-handbook) using Review Manager 5.2 [15].

### Data Synthesis and Statistical Analysis

Based on the literature and clinical physicians’ opinion, we defined the outcome measures as follows: (1) primary outcomes: stroke incidence, safety index-any bleeding without intracranial hemorrhage; (2) secondary outcomes: all-cause death, ischemic stroke, cerebral/intracranial hemorrhage, and TIA.

We calculated the relative risk (RR) for dichotomous data. Firstly, the analysis was performed with a fixed-effect model and the heterogeneity was considered significant if the χ^2^ test was significant (P value < 0.10) or the I^2^ > 50.00%.Then If we fond that the heterogeneity was significant, we further continued to analyze the clinical heterogeneity using the subgroup analysis. Among the five included trials, some followed the patients for relatively long time periods in clinical practice (>90 days follow-up), while others for a short time with certain days reported (≤90 days). After consulting the clinicians on how to report our results more precisely and reduce the heterogeneity, we analyzed them as short term [16, 17, 18] and long term [19, 20]. And the difference of race and region might also lead to the clinical heterogeneity. We compared the Asian and non-Asian using a subgroup analysis. Further to say, the participants’ hypertension, diabetes ratio and the dosages of clopidogrel and aspirin in the five studies varied to some degree among the studies, which could also affect the incidence of stroke. Publication bias was evaluated by visual examination of funnel plots and the Egger regression test. This meta-analysis was conducted using Stata13.1.

## Results

**Fig 1:**
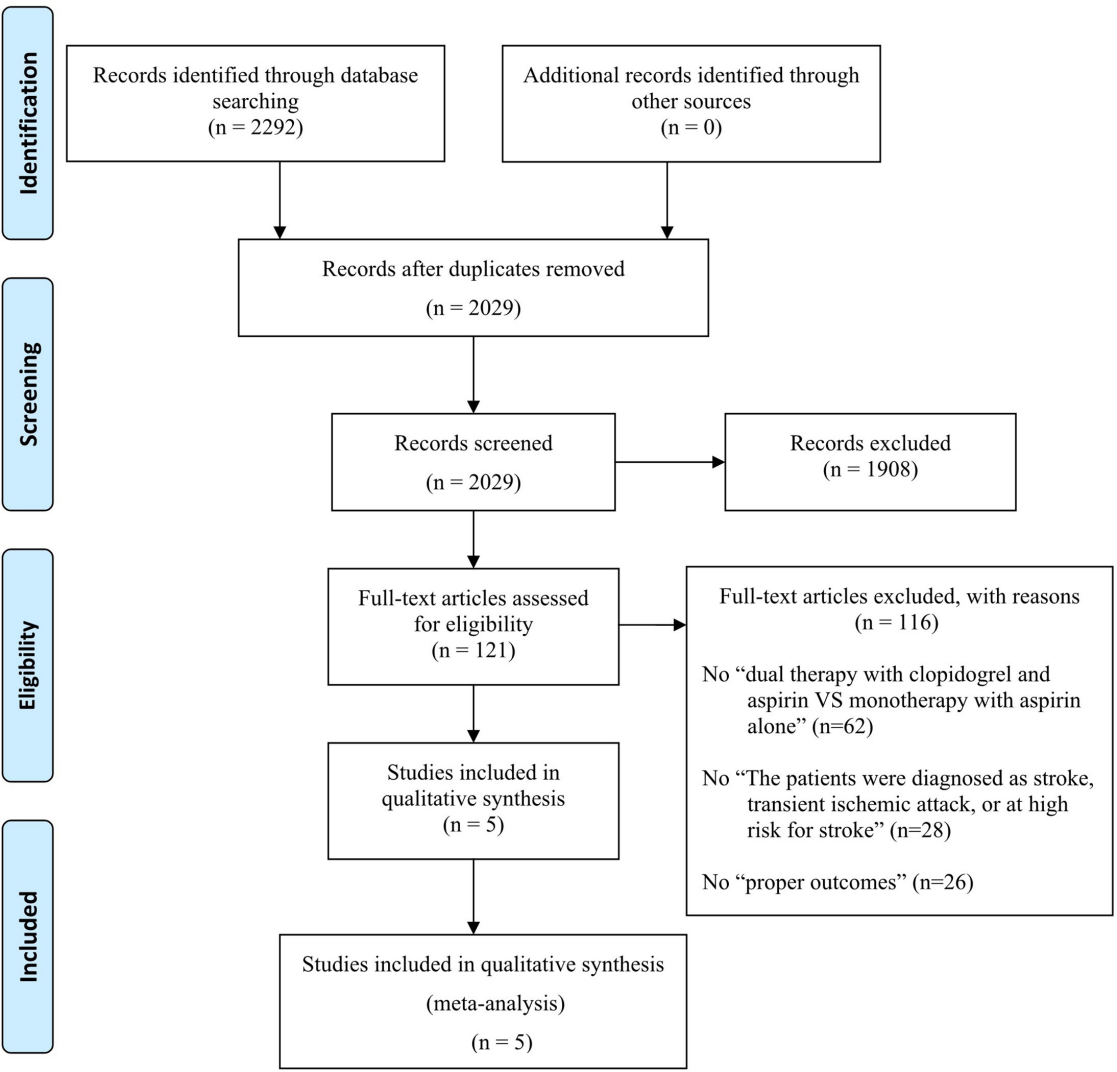
Flow diagram of study selection process.

A total of 2292 studies were identified through electronic searches, and 263 were excluded because of duplicates removed. Then 1908 studies were also excluded after reading the title and abstract. The remaining 121 studies were assessed by reading the full texts. The 116 full-text articles excluded, with reasons: 1.No “dual therapy with clopidogrel and aspirin VS monotherapy with aspirin alone” (62 articles); 2.No “The patients were diagnosed as stroke, transient ischemic attack, or at high risk for stroke” (28 articles); 3.No “proper outcomes” (26 articles). And there were 5 studies included in qualitative synthesis and meta-analysis finally. (The flow diagram of study selection process is shown in Fig 1.) Five studies with a total of 24,084 subjects met our inclusion criteria [16,17,18,19,20]. No additional RCTs were identified in a search of the included studies’ references. The characteristics of the included RCTs are summarized in Table 1(Characteristics of 5 Studies Included into Meta-Analysis) and Table 2(Table 2.The outcomes of each study). The studies varied with regard to author, publication year, sample size, baseline participant characteristics, treatment dosage, and treatment duration. All five studies concluded that dual therapy was more efficacious. All of the studies compared mono- and dual therapy, but one included two additional treatment groups [16]. Four studies that were registered on clinicaltrial.gov provided the complete results, and the fifth was registered with the Centre for Clinical Trials at the Chinese University of Hong Kong. The registration numbers of these experiments are ClinicalTrials.gov (NCT00109382) [16], number CUHK_CCT00164 [17], ClinicalTrials.gov (NCT00979589) [18], ClinicalTrials.gov (NCT00059306) [19],and ClinicalTrials.gov (NCT00050817) [20] respectively.

**Table 1:** Characteristics of 5 Studies Included into Meta-Analysis.

	N Patients (Dual VS Mono)	Mean Age, y/o		Men%		Previous stroke		Previous TIA		Hypertension %		Diabetes %		Dosage		Average follow-up
		Dual	Mono	Dual	Mono	Dual	Mono	Dual	Mono	Dual	Mono	Dual	Mono	Dual	Mono	
**Kennedy J2007** ^**16**^	193 (98 VS 95)	68.90	69.80	53.00	44.00	7/98	9/95	14/98	17/95	57.90	57.90	9.20	6.30	Aspirin 162mg+ clopidogrel 75 mg	Aspirin 81–162 mg	3 months
**Wong KSL2010** ^**17**^	98 (46 VS 52)	59.20	56.40	78.00	77.00	-	-	-	-	60.00	69.00	46.00	31.00	Clopidogrel 300 mg first day then 75 mg; aspirin 75–160 mg	Aspirin 75–160 mg	7 days
**Wang Y2013** ^**18**^	5170 (2584 VS 2586)	62.00	62.00	67.00	65.00	516/2584	517/2586	94/2584	80/2586	66.40	65.10	21.30	21.00	Aspirin 75–300 mg+ clopidogrel 75 mg	Aspirin 75–300 mg	3 months
**Bhatt DL2006** ^**20**^	15603 (7802 VS 7801)	64.00	64.00	70.00	70.00	1942/7802	1895/7801	938/7802	926/7801	73.30	73.90	42.30	41.70	Aspirin 75–162mg+ clopidogrel 75 mg	Aspirin 75–162 mg	28 months
**Benavente OR2012** ^**19**^	3020 (1517 VS 1503)	63.00	63.00	62.00	64.00	-	-	-	-	76.00	74.00	35.00	38.00	Aspirin 325mg+ clopidogrel 75 mg	Aspirin 325 mg	41 months

N; number; Dual: Dual therapy with clopidogrel and aspirin; Mono: monotherapy with aspirin alone; y/o: years old.

**Table 2:** The Outcomes of Each Study.

	The outcome: Stroke incidence (Events/Total, n/N)		The outcome: All-cause death (Events/Total, n/N)		The outcome: Ischemic stroke (Events/Total, n/N)		The outcome: Cerebral/intracranial hemorrhage (Events/Total, n/N)		The outcome: TIA (Events/Total, n/N)		The outcome: Any bleeding without intracranial hemorrhage (Events/Total, n/N)	
	Dual group	Mono group	Dual group	Mono group	Dual group	Mono group	Dual group	Mono group	Dual group	Mono group	Dual group	Mono group
**Kennedy J 2007** ^**16**^	5/98	9/95	-	-	-	-	-	-	-	-	-	-
**Wong KSL 2010** ^**17**^	0/46	1/52	-	-	0/46	2/52	-	-	2/46	1/52	2/46	0/52
**Wang Y 2013** ^**18**^	212/2584	303/2586	10/2584	10/2586	204/2584	295/2586	8/2584	8/2586	39/2584	47/2586	60/2584	41/2586
**Bhatt DL 2006** ^**20**^	150/7802	189/7801	371/7802	374/7801	132/7802	163/7801	26/7802	27/7801	-	-	320/7802	222/7801
**Benavente OR 2012** ^**19**^	125/1517	138/1503	113/1517	77/1503	100/1517	124/1503	21/1517	13/1503	28/1517	39/1503	87/1517	42/1503

Dual: Dual therapy with clopidogrel and aspirin;Mono: monotherapy with aspirin alone.

**Fig 2:**
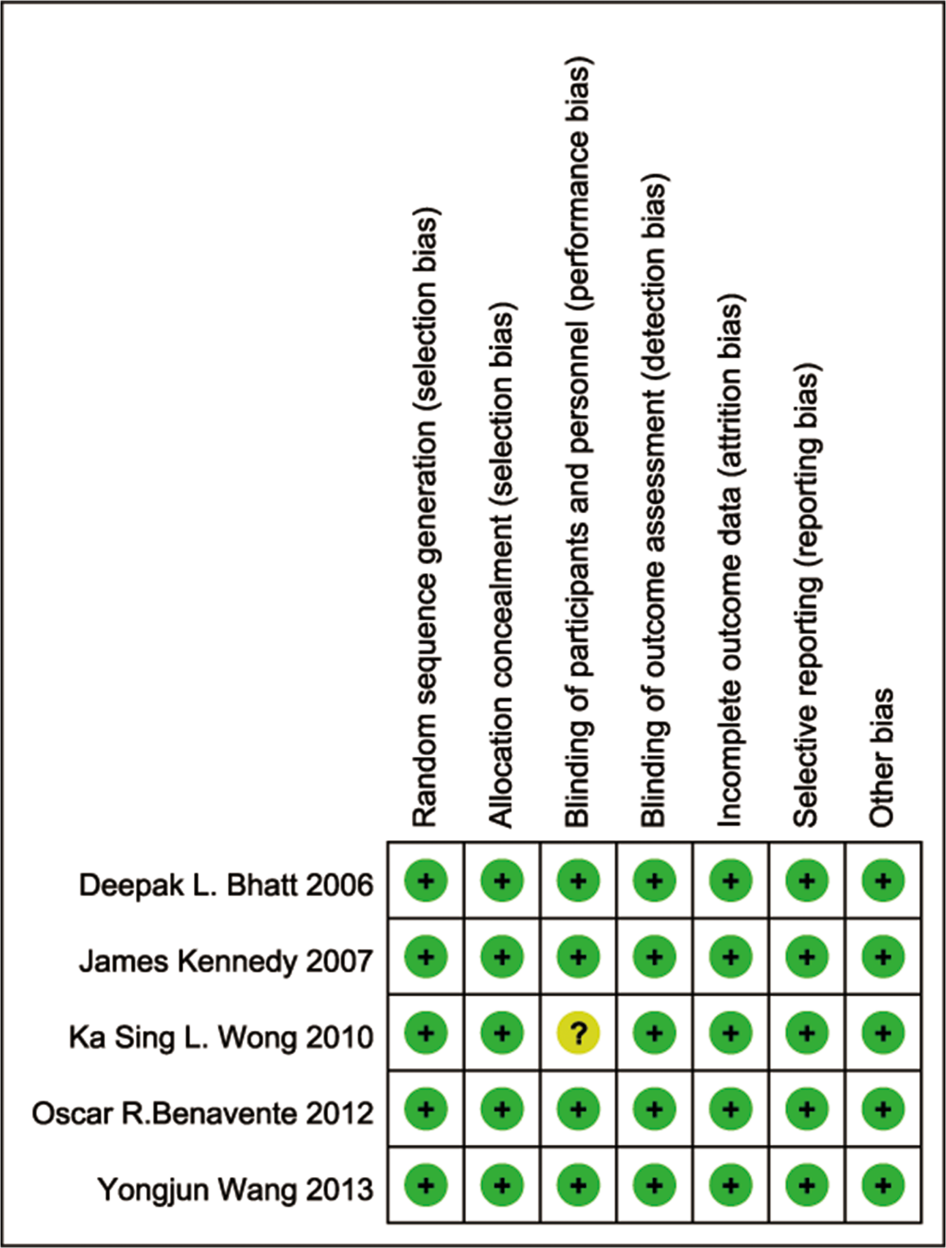
Risk of bias summary for each included study.

The risk of bias in the included studies is graphically summarized in Fig 2. Only one study had an unclear risk of bias for blinding based on a lack of reported information for patient blinding [17]. The other studies all had low risks of bias for all of the assessed items.

### Overall outcomes

**Fig 3:**
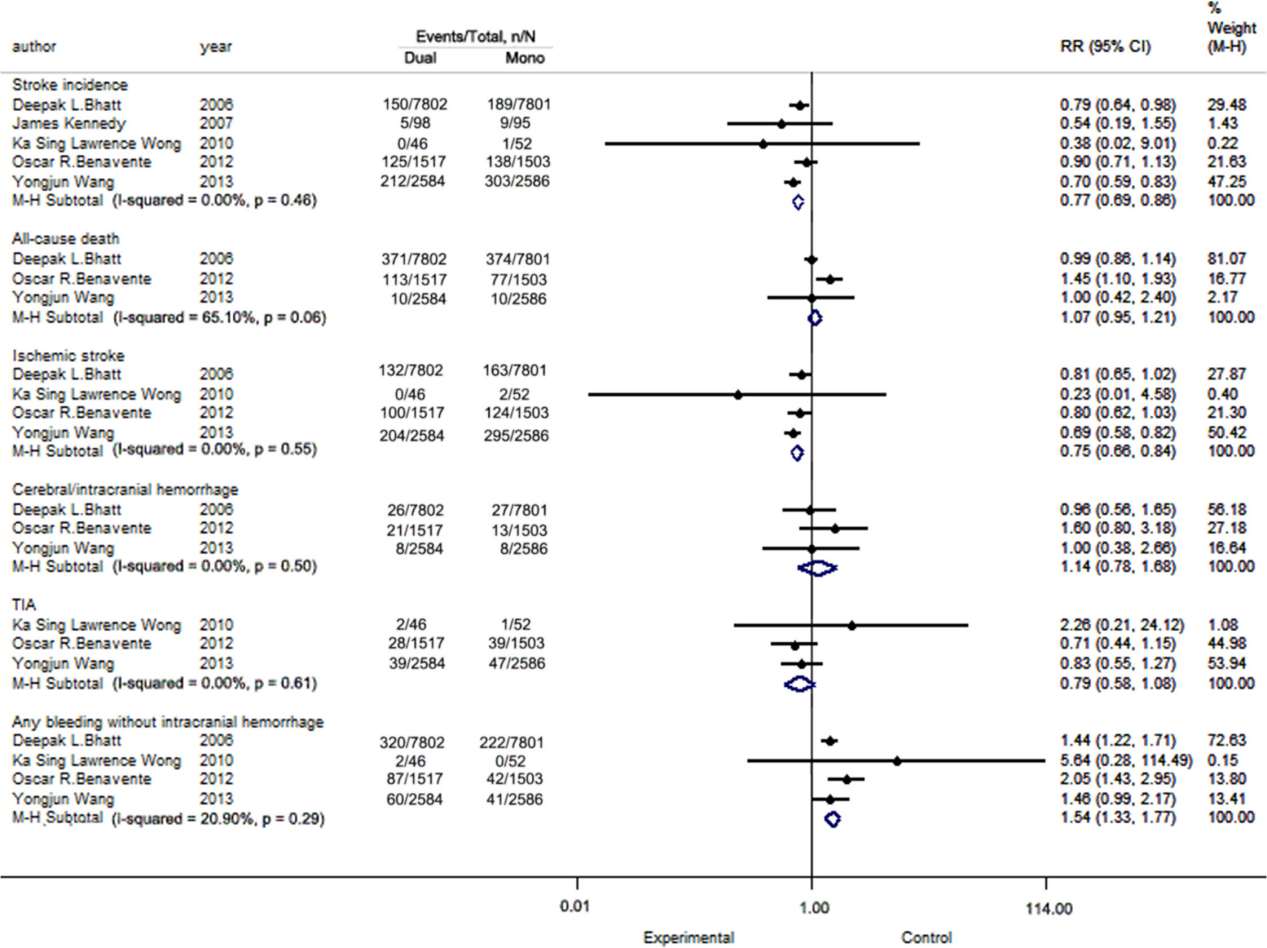
Pooled analysis for all study.

The details of the outcomes are presented in Fig 3.

#### Stroke incidence

All included trials reported this outcome. The statistical values for heterogeneity were P = 0.46, I^2^ = 0.00%. We obtained the following RR = 0.77, 95% confidence interval (CI): 0.69–0.86, P<0.05,which indicated a statistical significant difference between mono- and dual therapy. Clopidogrel and aspirin dual therapy is better than aspirin alone.

#### All-cause death

Three RCTs (23,793 patients) reported this outcome. The test of heterogeneity yielded a p value of 0.06 and an I^2^ value of 65.10%, which indicated high heterogeneity. We obtained the following RR = 1.07, 95% CI: 0.95–1.21, P = 0.29, which indicated no statistical significance between these two groups.

#### Ischemic stroke

Four trials (23,891 subjects) reported outcomes for ischemic stroke. The heterogeneity results were P = 0.55, I^2^ = 0.00%. After analysis, the results were RR = 0.75, 95% CI: 0.66–0.84, P <0.05, which indicated statistically significant difference between mono- and dual therapy.

#### Cerebral/intracranial hemorrhage

Three studies reported outcomes for cerebral/intracranial hemorrhage. No heterogeneity was found among these trials (P = 0.50, I^2^ = 0.00%). The results were RR = 1.14, 95% CI: 0.78–1.68, P = 0.50, indicating no statistical differences.

#### TIA

Three trials with data from 8,288 participants were pooled for this outcome. The results indicated no heterogeneity (P = 0.61, I^2^ = 0.00%), and the result (RR = 0.79, 95% CI: 0.58–1.08, P = 0.14) was not statistically significant.

#### Safety index: Any bleeding without intracranial hemorrhage

Four of the trials provided data for this outcome, and no heterogeneity was found (P = 0.29, I^2^ = 20.90%). However, the pooled results indicated that the addition of clopidogrel to aspirin may higher the risk of hemorrhage compared with aspirin alone (RR = 1.54, 95% CI: 1.33–1.77, P < 0.05).

### Subgroup Analysis and Sensitivity Analysis

Among the five included trials, some followed the patients for relatively long time periods in clinical practice (>90 days follow-up), while others only assessed the subjects for a short time with certain days reported (≤90 days). After consulting the clinicians on how to report our results more precisely, we analyzed them as short term (≤90 days) [16, 17, 18] and long term (>90 days follow-up) [19, 20].

### The analysis of short-term

**Fig 4:**
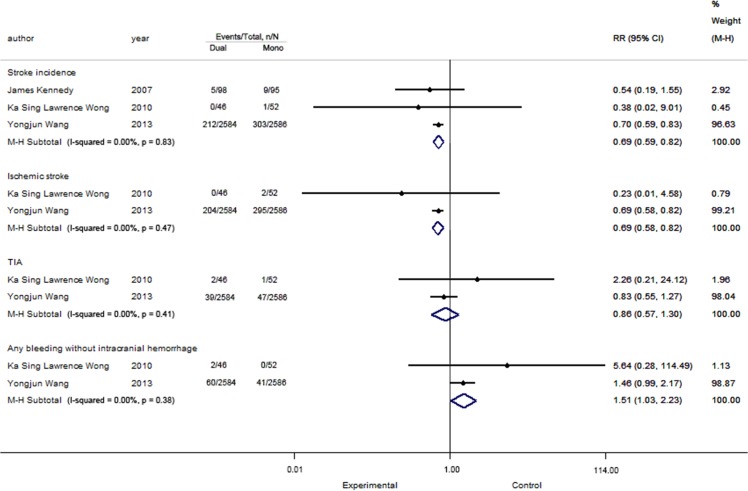
The analysis of short-term.

The analysis of short-term is depicted in Fig 4.

#### Stroke incidence

All three short-term studies reported this outcome and included data from 5,461 patients. The result showed that the patients who took both aspirin and clopidogrel had lower risk for stroke than those who took aspirin alone (RR = 0.69, 95% CI: 0.59–0.82, P < 0.05). No heterogeneity was found (I^2^ = 0.00%, P = 0.83).

#### Ischemic stroke

Two studies with 5,268 patients were included, and no heterogeneity was found (I^2^ = 0.00%, P = 0.47). The data showed a significant difference regarding the outcome of ischemic stroke (RR = 0.69, 95% CI: 0.58–0.82, P < 0.05).

**TIA.** Two studies with 5,268 patients were included, and no heterogeneity was found (I^2^ = 0.00%, P = 0.41). The data (RR = 0.86, 95% CI: 0.57–1.30, P = 0.47) showed no significant differences between the two groups in the short-term follow-up.

#### Safety index: Any bleeding without intracranial hemorrhage

Two studies with 5,268 patients were included, and no heterogeneity was found (I^2^ = 0.00%, P = 0.38). The data (RR = 1.51, 95% CI: 1.03–2.23, P = 0.04) showed a significant difference between the two groups. Dual therapy had a higher risk of bleeding than monotherapy.

### The analysis of long-term

**Fig 5:**
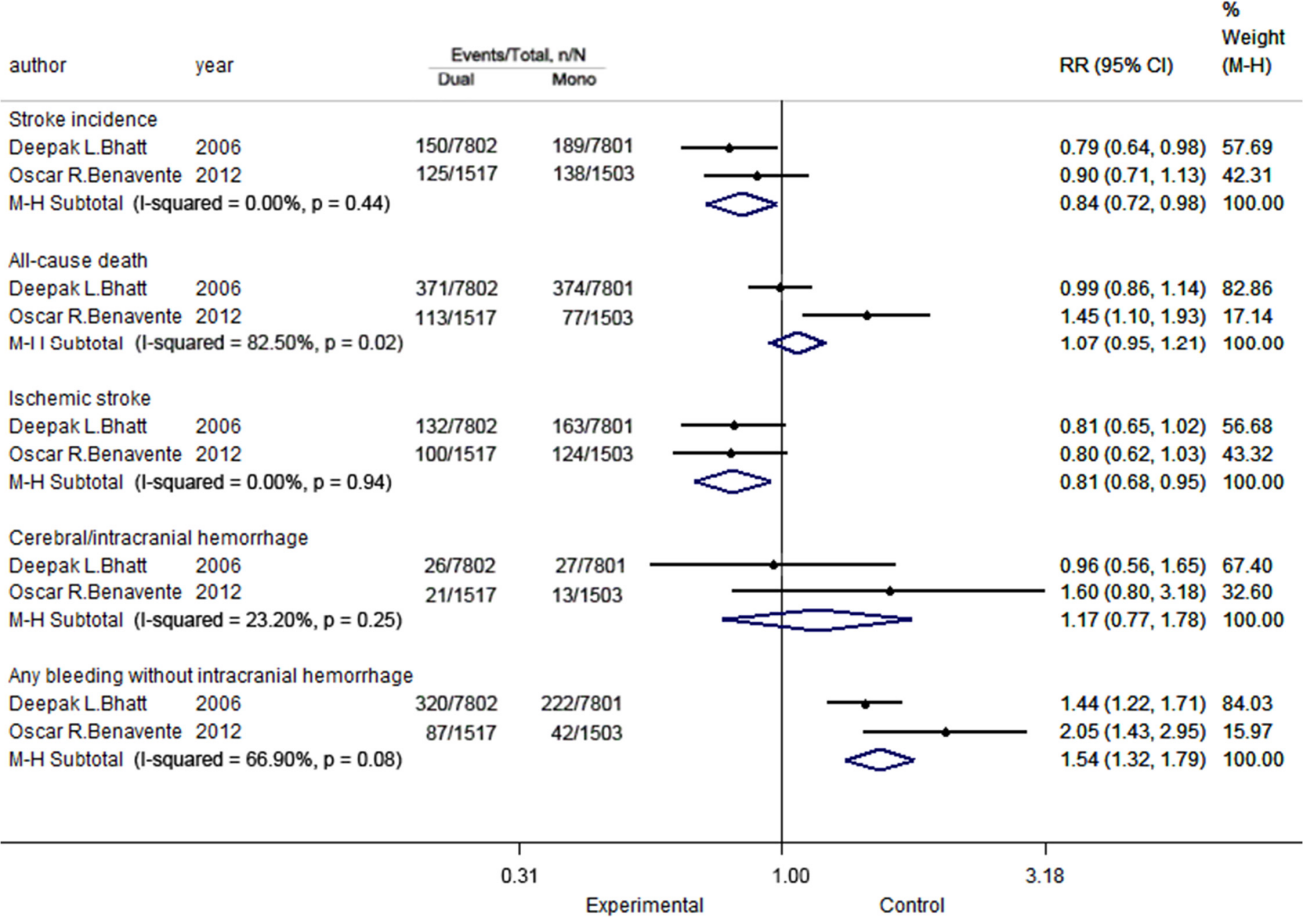
The analysis of long-term.

The analysis of long-term is depicted in Fig 5.We did not pool the data for TIA because only one study reported this outcome [19] in this section.

#### Stroke incidence

Two studies of 18,623 patients were included, and no heterogeneity was found for stroke incidence (I^2^ = 0.00%, P = 0.44). An analysis of the data revealed a significant difference between the two groups in the long-term follow-up. Dual therapy was associated with a low incidence of stroke (RR = 0.84, 95% CI: 0.72–0.98, P = 0.03).

#### All-cause death

Two studies with 18,623 patients were included, and significant heterogeneity was found (I^2^ = 82.50%, P = 0.02); however, the data (RR = 1.07, 95% CI: 0.95–1.21, P = 0.28) showed no significant difference between the two groups for the long-term follow-up.

#### Ischemic stroke

The same study and patients were included as for the former outcomes. No heterogeneity was found (I^2^ = 0.00%, P = 0.94). The data (RR = 0.81, 95% CI: 0.68–0.95, P = 0.01) showed significant difference between the two groups in the long-term follow-up.

#### Cerebral/intracranial hemorrhage

Two studies with 18,623 patients were included, and no heterogeneity was found (I^2^ = 23.20%, P = 0.25). The data (RR = 1.17, 95% CI: 0.77–1.78, P = 0.46) showed no significant difference between the two groups in the long-term follow-up.

#### Safety index: Any bleeding without intracranial hemorrhage

Two studies that assessed 18,623 patients were included, and heterogeneity was found (I^2^ = 66.90%, P = 0.08). The data was assessed, which showed significant differences between the two groups with regard to long-term follow-up (RR = 1.54, 95% CI: 1.32–1.79, P<0.05).

As written above, heterogeneity was found for several outcomes including “all-cause death on overall outcomes” and “all-cause death, safety index: any bleeding without intracranial hemorrhage on the analysis of long-term”. Therefore, we performed further analysis and wanted to find some reasons for the heterogeneity (details are as follows).

The differences of race and region might lead to the clinical heterogeneity and these patients were from non-Asian (Canada)[16], Asia(Hong Kong, Singapore, China, Thailand, and Malaysia)[17], Asia(114 centers in China)[18], non-Asia (North America, Latin America, and Spain)[19], Asia and non-Asian (32 countries and 768 sites)[20] respectively. So we compared the Asian and non-Asian using a subgroup analysis: (1) The result of Asian group [18,20] of all-cause death on overall outcomes was that no heterogeneity was found (I^2^ = 0.00%, P = 0.98). But the data (RR = 0.99, 95% CI: 0.86–1.14, P = 0.91) showed no significant difference. (2) The result of non-Asian group [19, 20] of all-cause death on overall outcomes was that heterogeneity was still found (I^2^ = 82.50%, P = 0.02). The data (RR = 1.07, 95% CI: 0.95–1.21, P = 0.28) showed no significant difference. (3) All-cause death and safety index: any bleeding without intracranial hemorrhage on the analysis of long-term these two indexes only included two studies respectively, so they could not be further analyzed in this way. Above all, the difference of race and region might be one of the reasons for high heterogeneity. Further to say, the participants’ hypertension, diabetes ratio and the dosages of clopidogrel and aspirin in the five studies varied to some degree among the studies, which could also affect the incidence of stroke.

### Publication Bias

The funnel plots of stroke incidence, all-cause death, ischemic stroke, cerebral/intracranial hemorrhage, TIA, safety index: any bleeding without intracranial hemorrhage in overall outcomes (S1A, S1B, S1C, S1D, S1E and S1F Fig) were approximately symmetrical, indicating the absence of significant publication bias. The Egger regression test suggested no obvious publication bias for the outcomes in overall outcomes: stroke incidence (P = 0.72>0.05, S2A Fig), all-cause death (P = 0.72>0.05, S2B Fig),ischemic stroke (P = 0.74>0.05, S2C Fig), cerebral/intracranial hemorrhage (P = 0.87>0.05, S2D Fig), TIA (P = 0.39>0.05, S2E Fig), safety index: any bleeding without intracranial hemorrhage (P = 0.31>0.05, S2F Fig).

## Discussion

Because of its high incidence rate, stroke is a major concern worldwide [21]. It was reported that almost 7 million people receive life-long antiplatelet treatment to prevent stroke. Therefore, we need to have a better understanding of the findings of published studies to inform clinical practice.

Kennedy et al. [16] reported that patients were at high risk of stroke immediately after TIA or minor stroke and that this might be reduced by using clopidogrel in addition to aspirin. The hemorrhagic risks of the combination of aspirin and clopidogrel do not seem to offset this potential benefit. In 2010, Wong et al. [17] published data that showed that combination therapy with clopidogrel and aspirin was more effective than aspirin alone in reducing microembolic signals in patients with predominantly intracranial symptomatic stenosis. Recently, a large-scale RCT undertaken by Wang and colleagues [18] indicated that for patients with TIA or minor stroke who could be treated within 24 hours after symptom onset, the combination of clopidogrel and aspirin was superior to aspirin alone for reducing the risk of stroke in the first 90 days and did not increase the risk of hemorrhage. The SPS3 Investigators [19] reported that among patients with recent lacunar strokes, the addition of clopidogrel to aspirin did not significantly reduce the risk of recurrent stroke but did significantly increase the risks of bleeding and death. Bhatt and colleagues [20] reported that there was a benefit of clopidogrel treatment in patients with symptomatic atherothrombosis and a suggestion of harm in patients with multiple risk factors. Overall, clopidogrel plus aspirin was not significantly more effective than aspirin alone in reducing the rate of myocardial infarction, stroke, or death from cardiovascular causes. Given the variability in the published results, we were interested in clarifying whether dual therapy is more efficacious than monotherapy and if it led to adverse effects.

After pooling data from relevant RCTs, we confirmed that dual therapy was superior in preventing stroke in both the short and long terms, and the effect of dual therapy seems to be more obvious for short-term use. However, a higher risk of bleeding without intracranial hemorrhage was found, which lowers our confidence in this dual therapy. For long-time use, the risk of bleeding is even higher. Nevertheless, we also found that the cerebral/intracranial hemorrhage rates were not significantly different.

In the 2011 guideline published by AHA/ASA [22], it was recommended that the addition of clopidogrel to aspirin should not be regarded as a routine therapy. However, this point was amended in the most recent guideline, and they recommended that for patients with recent stroke or TIA (within 30 days) attributable to severe stenosis (70–99%) of a major intracranial artery, the addition of clopidogrel (75 mg/d) to aspirin for 90 days might be reasonable. The results of this meta-analysis provide a better understanding for this modification to the guideline.

## Strengths and limitations

Our analysis included the high-quality, multi-center CLAIR, FASTER, and SPS3 studies. Furthermore, some of them included a large number of participants, which increased our confidence in our pooled results and its further use. Despite this strength, the participants’ race and region, hypertension, diabetes ratio and the dosages of clopidogrel and aspirin in the five studies varied to some degree among the studies, which may have affected the incidence of stroke leading to the high heterogeneity. We also took relatively strict standards about the inclusion and exclusion criteria, which led to all studies using Chinese language that can not meet our standards. And we also hoped these selected articles should have standard experimental registration numbers (like one study in our manuscript “Fast assessment of stroke and transient ischaemic attack to prevent early recurrence (FASTER): a randomised controlled pilot trial” has a ClinicalTrials.gov number, NCT00109382 [16]). There were only five studies in our study, so the few numbers of this study were also one of the limitations in our research.

In conclusion, dual therapy could be a good alternative for stroke patients and high-risk populations, and the effect of dual therapy seems to be more obvious for short-term stroke prevention. However, we should consider the cost and patient status while weighing the benefits and adverse effects of this dual therapy.

## Supporting Information

S1 Fig The funnel plots of indexes in overall outcomes respectively. (TIF)

S2 Fig The Egger regression tests of indexes in overall outcomes respectively. (TIF)
