# Supplementary figures and images for: Clopidogrel and Aspirin versus Aspirin Alone for Stroke Prevention: A Meta-Analysis
Source: PLoS One. 2015 Aug 13;10(8):e0135372. doi: 10.1371/journal.pone.0135372 (PMC4536208; doi:10.1371/journal.pone.0135372)

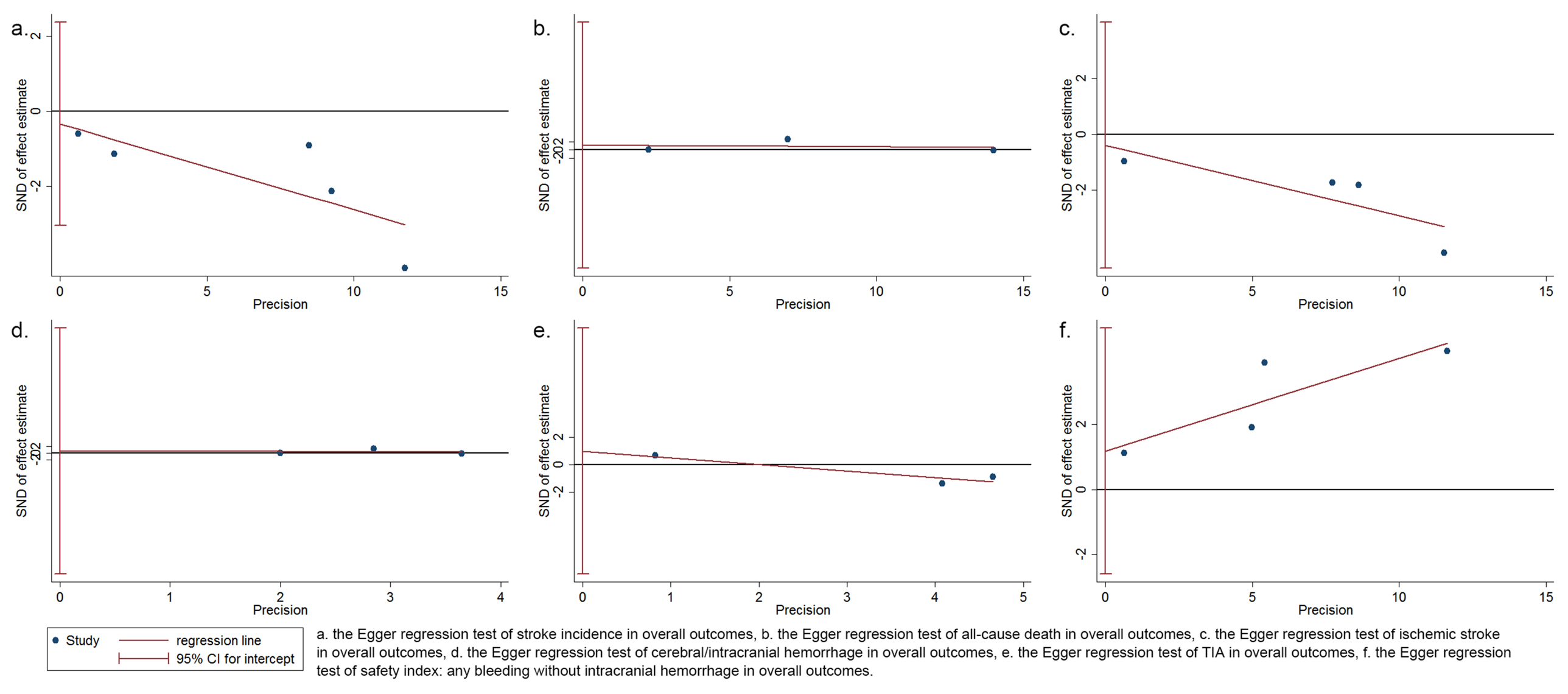

Supplement: S2 Fig — (TIF) [file pone.0135372.s002.tif]
